# PvaxDB: a comprehensive structural repository of *Plasmodium vivax* proteome

**DOI:** 10.1093/database/bay021

**Published:** 2018-03-14

**Authors:** Ankita Singh, Rahul Kaushik, Himani Kuntal, B Jayaram

**Affiliations:** 1 Department of Bioinformatics, Banasthali Vidyapith, Banasthali 304022, Rajasthan, India; 2 Supercomputing Facility for Bioinformatics and Computational Biology, IIT Delhi, Delhi, India; 3 Kusuma School of Biological Sciences, IIT Delhi, Delhi, India; 4 Department of Chemistry, Indian Institute of Technology Delhi, Hauz Khas, New Delhi 110016, Delhi, India

## Abstract

The severity of malaria caused by *Plasmodium vivax* worldwide and its resistance against the available general antimalarial drugs has created an urgent need for a comprehensive insight into its biology and biochemistry for developing some novel potential vaccines and therapeutics. *P.vivax* comprises 5392 proteins mostly predicted, out of which 4211 are soluble proteins and 2205 of these belong to blood and liver stages of malarial cycle. Presently available public resources report functional annotation (gene ontology) of only 28% (627 proteins) of the enzymatic soluble proteins and experimental structures are determined for only 42 proteins *P. vivax* proteome. In this milieu of severe paucity of structural and functional data, we have generated structures of 2205 soluble proteins, validated them thoroughly, identified their binding pockets (including active sites) and annotated their function increasing the coverage from the existing 28% to 100%. We have pooled all this information together and created a database christened as PvaxDB, which furnishes extensive sequence, structure, ligand binding site and functional information. We believe PvaxDB could be helpful in identifying novel protein drug targets, expediting development of new drugs to combat malaria. This is also the first attempt to create a reliable comprehensive computational structural repository of all the soluble proteins of *P. vivax*.

**Database URL**: http://www.scfbio-iitd.res.in/PvaxDB

## Introduction

*Plasmodium vivax* is a widespread malarial pathogen among humans having 71 different mosquito species as vectors and trademarked among most lethal human pathogenic species (1–3). Recent statistics report 35% of the world population to be at a risk of getting infected with *P. vivax*, with an annual estimate of 80–300 million cases (4–6). The burden of this disease may be reduced via a better understanding of the biology and biochemistry of the parasite and by developing potential vaccines, diagnostics and therapeutics (7–9). The emergence of drug resistance to available drugs (primaquine and chloroquine) and lack of commercially available vaccines has created an alarming situation to combat *P. vivax* caused malaria (10–14) thus emphasizing the necessity for the identification of some novel drug targets, which could lead to development of new drugs via structure-based drug discovery endeavors (15–19). Structure-based drug discovery requires the availability of protein structures and unfortunately, only 1% of the *P. vivax* proteome (<50 structures) has experimentally known structural data, which is a major bottleneck for identifying novel drug targets and development of new drugs. The time consuming and expensive experimental approaches (20–25) are very unlikely to deliver structural information of the uncovered *P. vivax* proteome in the near future. Meanwhile, computational methods for protein structure prediction and function characterization have matured enough over the years to provide a promising supplement and thus may help in accelerating the initial phase of drug discovery (26–31). There are databases that have provided information on *Plasmodium* genus like PlasmoDB (32), which offers vast information on annotated genomes, transcription level evidences, proteomics evidences and so on for various species of malarial parasite. Notably, the structure level information is not provided in PlasmoDB intrinsically. Another database, named ModBase (33) provides structural information for at proteome level for several species. The structural information in ModBBase is generated by purely homology-based tool, Modeller and the reliability of these modeled structures declines in absence of suitable homolog protein or inaccurately identified homolog protein in low sequence identity with experimental structures.

Here, we have developed structural repository of 2205 soluble proteins with the main focus on proteins involved in blood and liver stages. The database furnishes extensive sequence information, which is known from literature and structure, ligand binding site and functional information developed as a part of this work. This structural databank could serve as an aid to identifying potential novel drug targets for designing and developing lead molecules in order to combat *P. vivax* caused malaria.

## Materials and methods

The development of PvaxDB is carried out in five different steps. In the process of compilation of the datasets, various tools/software/servers are implemented, which are thoroughly validated and widely accepted in their fields, to ensure reliability and a high level of confidence in the predictions. The overall process of sequence data collection, structure generation and validation, ligand binding site characterization and functional annotation is described below.

### Step 1: data extraction

The whole genome sequencing of *P.vivax* (strain Sal-I) resulted in identification of 5478 genes and 5392 proteins (genome assembly id: 22661). The latest data adopted from UniProtKB (34) contain 5389 proteins for *P. vivax* proteome (proteome id: UP000008333) with 4211 as soluble proteins and 1178 as membrane proteins, distributed over 14 chromosomes. Further the soluble proteins are classified into blood stage and liver stage proteins with the help of experimental information extracted from PlasmoDB (http://PlasmoDB.org), which stores the protein information at expression levels at different stages in primate host for various species of plasmodium parasite (32). A total of ∼2205 proteins represent blood and liver stages of *P. vivax* proteome in humans with 1755 and 59 proteins exclusively for blood and liver stages, respectively, along with a set of 491 proteins which represent both the stages. Here, we present a comprehensive sequence, structural and functional information for 2205 proteins of *P. vivax* which essentially covers all the proteins involved in its various pathogenic stages of asexual amplification in humans.

### Step 2: sequence level information compilation/prediction

All the protein sequences considered in the previous step (2205 in total) are processed to obtain different sequence dependent features, which include calculation of physico-chemical features, prediction of secondary structural features, protein homolog detection, prediction of structural modelability of proteins via structural difficulty (SD) index (35), and so on. The physico-chemical features include calculation of amino acid compositions, isoelectric points, molecular weights, aliphatic indices, aromaticity, instability indices, optical density and so on of the proteins. The secondary structural features include prediction of protein secondary structure via an improved in-house version of PSIPRED (36) and a further analysis in terms of number of helices, strands, coils, percentage of amino acid residues accounting for helices, strands and coils. The homolog detection is carried out via PSIBLAST (37) and the various parameters related to homology (e.g. sequence identity, sequence similarity, sequence coverage and number of gaps in alignment) are calculated for all the proteins. The physico-chemical, secondary structural and homology-based feature calculation is followed by the prediction of protein sequence modelability via SD index that provides insights into the possibility of a reliable tertiary structure prediction of the proteins. The success of protein structure prediction is believed to be a function of sequence similarity/identity of a query protein sequence with experimentally solved structure. However, this view can be misleading sometimes. SD index derives the modelability of a protein sequence on a scale of 0–100 from its physico-chemical, secondary structural and homology based features. A description of the features that are calculated is furnished in the help section of the databank and summarized in Table 1. Apart from these features, we also compiled sequence-based experimental data about the proteins wherever available.

**Table 1.** A brief summary of the various features compiled/predicted at protein sequence level for *Plasmodium vivax* proteome

Tool/Method	Brief description	Availability	CPU compute time per protein
UniProtKB	UniprotKB is a composite repository protein sequences and functional information.	www.uniprot.org	NA
PSIPRED	PSIPRED is an artificial neural network machine learning-based secondary structure prediction method.	www.bioinf.cs.ucl.ac.uk	15–20 min
SD index	Structural difficulty index predicts the modelability of a protein sequence into its tertiary structure.	www.scfbio-iitd.res.in	4–5 min
Other parameters	Other sequence features are computed via in-house programs based on parameters adopted from the literature.	www.scfbio-iitd.res.in	NA

### Step 3: structure level information compilation/prediction

The structural repository comprises structural information for all the sequences compiled in step 2. The protein tertiary structure prediction is carried out via three different state of the art methodologies viz. I-TASSER (38), RaptorX (39) and BhageerathH^+^ (40–43). I-TASSER implements multiple threading approach and generates full-length atomic models via fragment assembly and simulated annealing. RaptorX performs protein tertiary structure prediction via remote homolog detection and protein threading methods without performing conventional profile–profile matching. BhageerathH^+^ is an integrated software suite, which performs protein tertiary structure prediction via implementing *ab initio*/homology-based hybrid methodology as summarized in Table 2. The protein structure prediction is followed by an intensive quality assessment for all the predicted model structures via ProTSAV (44), which is an integrated metaserver consisting of Verify-3 D (45), Errat (46), Procheck (47), MolProbity (48), D2N (49), ProSA (50), dDFIRE (51), solvent accessible surface area (52) and so on as discussed in Supplementary material. The predicted model structures via all methodologies are made available to user along with a quality assessment report. ProTSAV evaluates the protein structures by considering the assessment parameters derived from experimental structures and predicted model structures of varying root mean square deviation (rmsds) (viz. 0–2 , 2–5 , 5–8  and beyond 8 A) as benchmarks and compares the corresponding parameters derived from input protein structure. This benchmarking enables ProTSAV metaserver to assess the input structure into any one of the rmsds classes. The structures scoring in 0–2 Å predicted rmsds (green region) are considered as high accuracy model structures. Similarly, structures scoring in 2–5 Å predicted rmsd (yellow region) are considered as medium accuracy model structure. The structures having predicted rmsds under 5 A (high and medium accuracy models) are useful in structure-based studies.

**Table 2.** A brief summary of the methods/tools used for features compilation/prediction at protein structure level along with their availability

Method	Brief description	Availability	CPU compute time per protein
I-TASSER	Fragment assembly and simulated annealing-based multiple threading approach for protein structure prediction.	www.zhanglab.ccmb.med.umich.edu	∼48–72 h
RaptorX	A remote homolog detection and threading-based proteins tertiary structure prediction method.	www.raptorx.uchicago.edu	∼5–6 h
BhageerathH^+^	An *ab initio*/homology-based integrated software suite for protein tertiary structure prediction.	www.scfbio-iitd.res.in/bhageerathH+	8–12 h
ProTSAV	Implements metaserver approach for extensive protein structure quality assessment.	www.scfbio-iitd.res.in/ProTSAV	2–3 min

### Step 4: tertiary structure-based ligand binding site prediction

Identification of ligand binding pockets of target proteins is considered as a fundamental requisite for structure based drug discovery (53, 54). Ligand binding regulates the biochemical function(s) of the target proteins. Further, a knowledge of the binding pockets helps in exploring mechanisms of molecular recognition and in functional characterization of proteins (55, 56). Considering the importance of ligand binding site information/detection/prediction, all the predicted model structures in PvaxDB, are subjected to ligand binding site prediction via some of the well-known and widely accepted softwares/tools, viz. LigSite (57), F-Pocket (58) and AADS (18). These softwares implement different approaches for ligand binding site prediction for a given protein tertiary structure. For instance, the LigSite captures surface-solvent-surface events via protein’s Connolly surfaces and identified pockets are ranked on the basis of the extent of conservation of surface residues involved. The F-pocket is based on Voronoi partition and alpha sphere theory and the pocket ranking is performed via partial least square fitting. The AADS methodology implements physicochemical features of functional groups lining the cavities on the protein surface for ligand binding site prediction as summarized in Table 3. In PvaxDB, top four ligand binding sites, as per individual software ranking, are provided and displayed with help of protein structure visualizer.

**Table 3.** A brief summary of ligand binding site identification/prediction tools used in PvaxDB for ligand binding site prediction of all the 6600 modeled structures

Method/Tool	Brief description	Availability	CPU compute time per protein
LigSite^csc^	A Connolly surface and the degree of conservation-based pocket identification tool on protein surface.	www.projects.biotec.tu-dresden.de	2–3 min
FPocket	A Voronoi partition and alpha sphere theory-based protein pocket (cavity) detection method.	www.bioserv.rpbs.univ-paris-diderot.fr	2–3 min
AADS	An automated active site detection and scoring methodology for identifying potential ligand binding site in proteins structures.	www.scfbio-iitd.res.in/dock/ActiveSite	2–3 min

### Step 5: sequence, structure and ligand site based function annotation

Exploring protein functions is very critical for understanding life at the molecular level (59, 60). With the help of precise terminology, the gene ontology consortium classified various functions of proteins into three categories, viz. molecular functions, biological processes and cellular components (61, 62). In PvaxDB, we performed protein function annotations using SIFTER (63) that implements a statistical approach using phylogenetic analysis for representing protein relationships, InterPro (64) performs protein sequence analysis and classification using prediction models or signatures compiled from different databases and LocTree3 (65), which is a support vector machine learning-based hierarchical system for searching proteins of experimental localizations and function prediction. Apart from these tools, based on potential ligand binding sites computed earlier, functional characterization is also performed by comparing them with experimental datasets using ProBiS tool (66). A brief summary of these tools/methods and their availability is provided in Table 4. The function annotation in terms of gene ontology by these individual methodologies for all the proteins is carried out and made available via PvaxDB webserver.

**Table 4.** A brief description of function prediction software used for functional characterization

Method/Tool	Brief description	Availability	∼Compute time per-protein
SIFTER	A statistical approach using phylogenetic analysis for representing protein relationships and functional characterization.	www.sifter.berkeley.edu	8–10 min
InterPro	Protein sequence analysis and classification via prediction models or signatures assembled from different databases.	www.ebi.ac.uk/interpro	5–6 min
LocTree3	A machine learning-based hierarchical system for experimental localizations and function prediction of proteins.	www.rostlab.org/loctree3	3–4 min
ProBiS	A protein structure surface conservation-based similar protein binding sites detection tool.	www.probis.cmm.ki.si	45–60 min

### PvaxDB database architecture

The back end of PvaxDB is built on MySQL and the web interface is created via implementation of PHP, JavaScript, HTML5 and AJAX technologies. The webpage access service is based on Apache. The PvaxDB implementation is summarized in Supplementary Figure S1. These technologies facilitate the users with convenient browsing through the PvaxDB by combining multiple queries/searches.

### How to use PvaxDB?

The information stored in PvaxDB can be explored through keywords individually or in combination (e.g. Protein Sequence, Chromosome Number, UniProt Identifier, Gene Ontology Terms, Pfam Identifier, InterPro Identifier, PubMed Identifier and Pvax Identifier) as summarized in Table 5.

**Table 5.** A summary of search keywords which can be used for a systematic and specific browsing of PvaxDB

Search keyword	Brief description	Example
Protein sequence	User can provide amino acid sequence in single letter code of desired protein. The output will result in a Pvax identifier corresponding to input sequence.	MDGGEDEGATEESIPVVILD….ANVL
Chromosome number	The genome of *P. vivax* consists of 14 chromosomes and user can browse the database for proteins corresponding to any of the chromosomes by selecting chromosome number(s).	Any chromosome number from 1 to 14
UniProt identifier	It is a unique identifier (six or ten letter alphanumeric string) assigned to each protein deposited in UniProt.	A5KAJ7
Protein name	User can explore the database with the help of protein names.	40S ribosome protein
Gene ontology (GO)	The GO terms are unique accession numbers of gene and gene product attributes across all species and can be used as search keywords in PvaxDB.	GO: 0000016
Protein family or Pfam identifier	Name of protein families or their Pfam identifiers can be used for exploring the databank. A Pfam identifier is a seven letter alphanumeric string.	Actin family PF00022
InterPro identifier	The InterPro database assigns each entry with a unique accession number in the form of an alphanumeric string starting with a prefix ‘IPR’	IPR000001
Pubmed identifier (PMID)	PMID is a unique identifier number assigned to each article record when it enters the PubMed system	18843361
Pvax identifier	Pvax identifier is a 10 letter unique alphanumeric string assigned to all proteins of *P. vivax* by PlasmoDB.	PVX_000750

**Figure 1.**
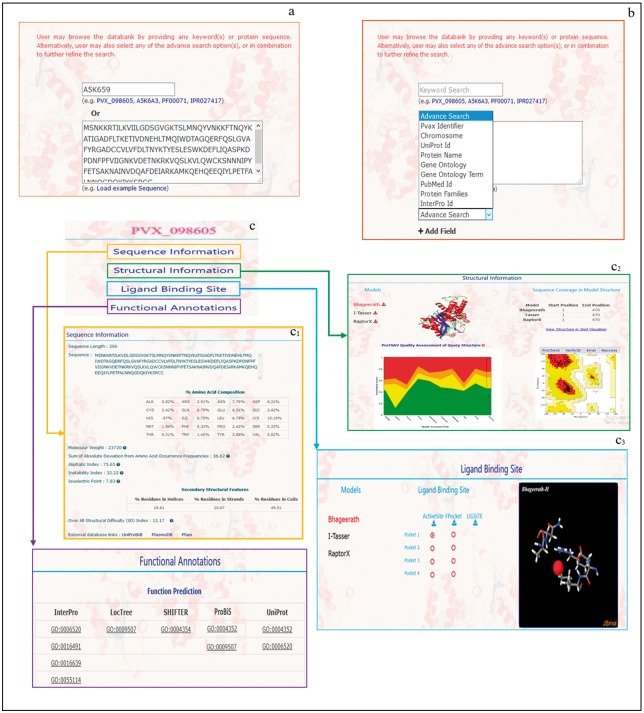
A depiction of different information provided in PvaxDB about sequence, structure, ligand binding site and function annotations.

The keyword(s) search returns the corresponding hits in the form of Pvax identifiers of protein(s). User may extract detailed information about any of the Pvax identifiers by clicking on them. The detailed information is categorized into four sections, viz. sequence information, structural information, ligand binding site information and functional annotation as discussed stepwise in Supplementary material and shown in Figure 1. A stepwise detailed tutorial is available in the help section of PvaxDB web interface.

## Discussion

The genome sequencing and transcriptome studies of *P. vivax* have furnished an extensive list of unexplored blood and liver stage proteins and identification of new protein druggable targets among these proteins may aid significantly in novel drug/vaccine development programs. However, protein structure-based drug discovery involves protein structures and unfortunately in case of *P. vivax*, a very small fraction of its proteome has experimental structural information, which restricts the novel drug target identification and development of more specific drugs.

Against this backdrop, we have developed the PvaxDB, a comprehensive structural repository which offers an extensive collection, curation, prediction and analysis of *P. vivax* proteome via some of the best state of the art methods in respective fields like protein structure prediction, quality assessment, ligand binding site identification and functional characterization. The methodologies adopted in PvaxDB required ∼63 h of extensive computation per protein on multiple processors for performing the described predictions/annotations. In PvaxDB, we have addressed blood and liver stage proteins (2205 proteins) of *P. vivax* for which the predictions/annotations are computed on a parallel implementation on multiple processors. Some of the major outcomes are summarized below.

**Figure 2.**
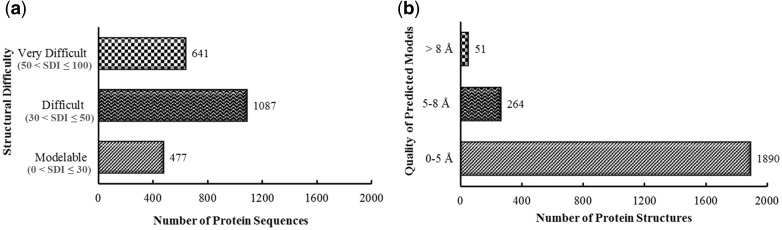
Pre-PvaxDB structural difficulty and post-PvaxDB quality assessment analysis. (**a**) Structural modelability based categorization of blood and liver stage soluble proteins of *P. vivax* proteome. (**b**) Categorization of predicted model structures of *P. vivax* based on an extensive quality assessment performed via ProTSAV.

**Figure 3.**
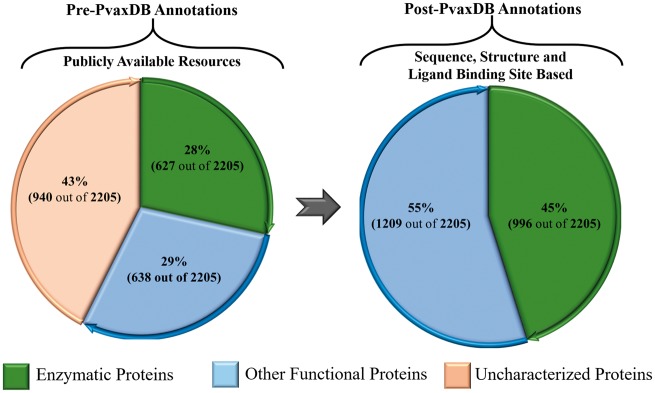
A depiction of functional characterization of *P. vivax* proteome. The left panel shows functional annotation of *P. vivax* proteome presently available through various public resources. The right panel shows functional annotations carried out in PvaxDB based on sequence, structural and ligand binding site information-based functional annotation.

1. The structural modelability characterization via structural difficulty index suggested that 1728 out of 2205 proteins were among difficult or very difficult to model (Figure 2a).
2. By providing 1890 proteins (86%) predicted as high and medium accuracy model structures, as evaluated through metaserver approach based extensive quality assessment, PvaxDB assures reliability of structural information provided (Figure 2b).
3. As compared to only 24 protein families out of total 1791 (1.3%) with available experimental structural information, PvaxDB offers structural and functional information of 1115 protein families out of the total of 1791 (62.2%) identified protein families in *P. vivax* proteome.
4. The functional information for soluble proteins of blood and liver stage is extended to 100%, based on phylogenetic analysis, signature pattern detection, experimental localization and identified ligand binding sites analysis in PvaxDB (Figure 3).

## Conclusion

In the current genomic era, it is very challenging to study and present the ever increasing data in a meaningful way. PvaxDB is a comprehensive structural repository to deliver to the scientific community extensive information regarding sequences, structures, ligand binding sites and functional annotations of *P.vivax* proteins on a single platform. The newly developed structural repository could be insightful for the parasite’s transmission process and interactions, which may further help in proposing and prioritizing novel protein targets for development of new drugs.

We intend to continuously update the information at PvaxDB in future releases.

## Supplementary data

Supplementary data are available at *Database* Online.

## Supplementary Material

Supplementary Data
