# Supplementary material for: PvaxDB: a comprehensive structural repository of Plasmodium vivax proteome
Source: Database (Oxford). 2018 Mar 14;2018:bay021. doi: 10.1093/database/bay021 (PMC5852996; doi:10.1093/database/bay021)
Supplement: Supplementary Data [file bay021_supp.docx]

**SUPPLEMENTARY INFORMATION**

**PvaxDB: A comprehensive structural repository of *Plasmodium vivax* proteome**

Ankita Singh^1, 2^, Rahul Kaushik^2, 3^, Himani Kuntal^1^, B. Jayaram^2, 3, 4*^

^1^Department of Bioinformatics, Banasthali Vidyapith, Banasthali, 304022, India,

^2^ Supercomputing Facility for Bioinformatics & Computational Biology, IIT Delhi, India,

^3^ Kusuma School of Biological Sciences, IIT Delhi, India,

^4^ Department of Chemistry, Indian Institute of Technology Delhi,

Hauz Khas, New Delhi, 110016, India

*Corresponding author


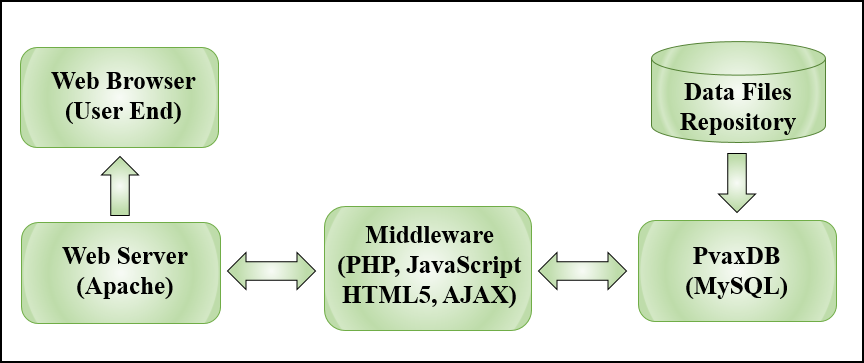
**Figure S1.** A depiction of PvaxDB architecture from data files repository to graphic user interface via MySQL, PHP, JavaScript, HTML5, AJAX and Apache.


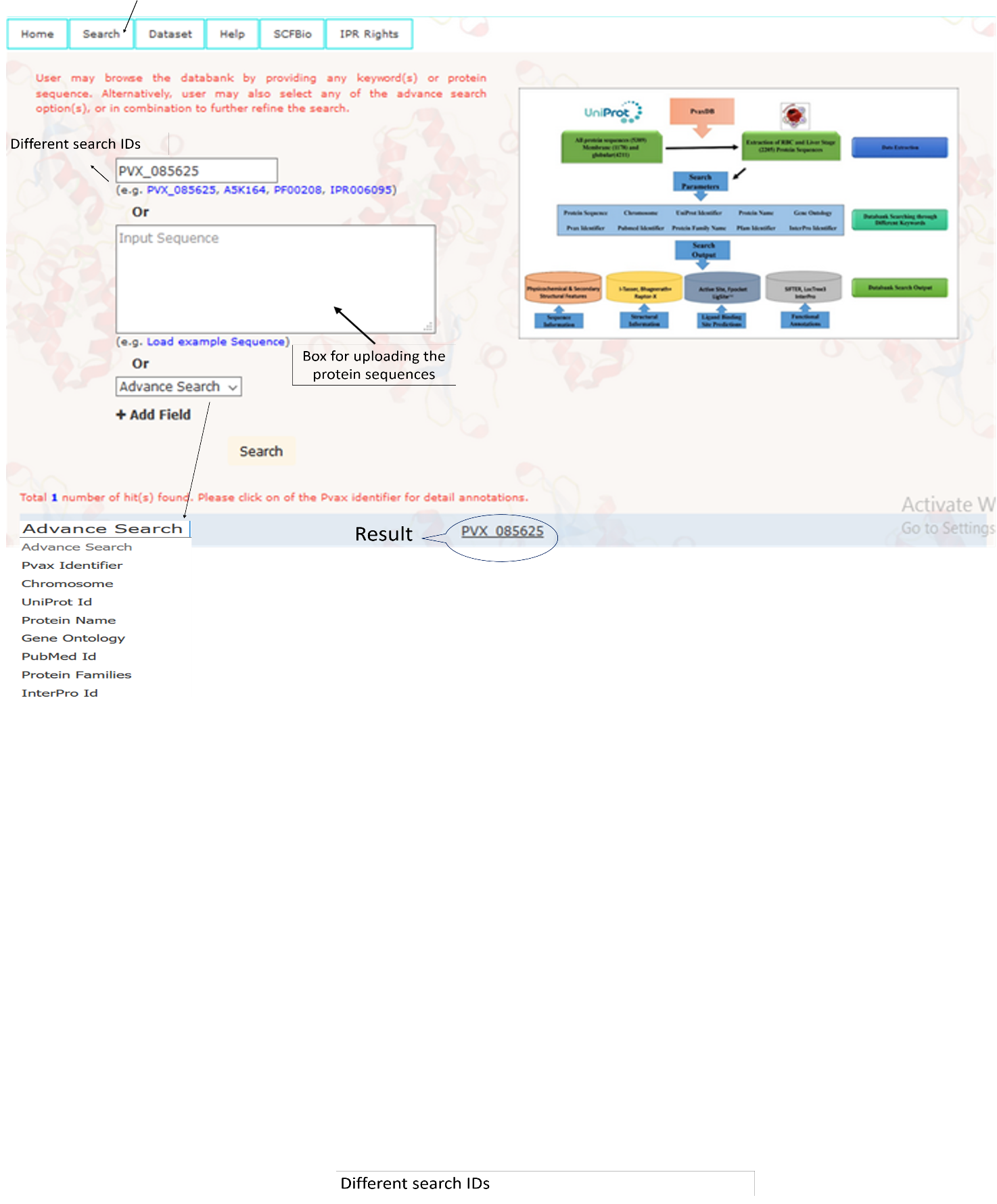
**Step I: How to Search PvaxDB**

**Figure S2.** A snapshot of PvaxDB webserver. Various key terms (e.g. Pvax Idetifier, Chromosome number, UniProt Identifier, PFam Identifier, etc.) for browsing PvaxDB are indicated.

PvaxDB provides Pvax Identifier(s) for searched terms (e.g. PVX_085625 in above case). Depending upon the search terms used the number of hits may be more than one also. For instance, if PFam Identifier ‘PF00208’ is used as search term, two hits are provided as results (PVX_085005 and PVX_085625). Similarly, if ‘Chromosome 1’ is selected in advanced search option, a total of 64 hits are provided as results. User may click on any of the hits to get detailed information about the protein. The various advance features can be used individually or in combination for browse the PvaxDB webserver.

**Step II: Analyzing the Result Page**


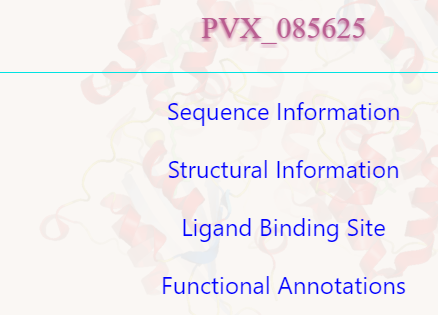
Clicking on the hits related to search term, the user is directed to new tab in the browser. The new tab shows for options viz. Sequence Information, Structural Information, Ligand Binding Site and Functional Annotations as shown in Figure S3. Clicking on any of the options displays corresponding information related to the selected protein. Here, the result page for PVX_085635 is explained in detail for better clarity.

**Figure S3.** Result page of PvaxDB webserver showing links for different information for searched protein (e.g. PVX_085625).

**Step III. Sequence Information**

The sequence information expands to provide various information derived/computed/predicted from protein sequence alone as shown in Figure S4. Various sequence based features provided under sequence information are briefly discussed ahead.

- **Sequence Length**: This indicates the number of amino acid residues in protein sequence (for PVX_085625, the sequence length is 470 amino acid residues)
- **Molecular Weight:** Molecular weight is calculated for the protein sequence by adopting the parameters defined in [John M. Walker (ed): The Proteomics Protocols Handbook, Humana Press (2005)](http://www.springer.com/life+sciences/biochemistry+%26+biophysics/book/978-1-58829-343-5), pp. 571-607.
- **Sum of Absolute Deviation from Amino Acid Occurrence Frequencies**: The amino acid occurrence frequencies are computed from the protein sequence and compared with the amino acid occurrence frequencies adopted from UniProt database (Table S1).

**Table S1.** The occurrence frequencies of amino acids adopted from UniProtKB

| Amino Acid | Frequency | Amino Acid | Frequency |
| --- | --- | --- | --- |
| Alanine | 0.088 | Leucine | 0.098 |
| Arginine | 0.056 | Lysine | 0.052 |
| Asparagine | 0.040 | Methionine | 0.024 |
| Aspartate | 0.054 | Phenylalanine | 0.040 |
| Cysteine | 0.013 | Proline | 0.048 |
| Glutamine | 0.039 | Serine | 0.068 |
| Glutamate | 0.062 | Threonine | 0.056 |
| Glycine | 0.071 | Tryptophan | 0.013 |
| Histidine | 0.022 | Tyrosine | 0.030 |
| Isoleucine | 0.0574 | Valine | 0.068 |

- **Aliphatic Index**: Relative volume occupied by aliphatic amino acid residues in protein sequence define the aliphatic index of proteins. These residues includes alanine, valine, leucine and Isoleucine. High aliphatic index is assumed to impart thermal stability to globular proteins.
- **Instability Index**: An estimation about the stability of protein can be derived from its instability index. Proteins with lower instability index (< 40) are considered as stable and vice-versa.
- **Isoelectric Point**: The pH at which the net charge of a protein is zero is considered as its isoelectric point (represented pI). The parameters for calculating pI are adopted from <https://web.expasy.org/compute_pi>.
- **Secondary Structural Features**: For all the protein sequences the secondary structure prediction is performed by PSIPRED and percentagage secondary structural content is provided to user.
- **Structural Difficulty (SD) Index:** The structural difficulty index accounts for physico-chemical, secondary structural and homology feature for calculating the ease of modelability of a protein sequence on a scale of 0 to 100 where score below 30 is considered as Modelable Zone, score from 30 – 50 is considered as Difficult Zone and score beyond 50 is considered as ‘Very Difficult Zone’. Detail regarding SD Index may be referred to <http://scfbio-iitd.res.in/SDIndex>.
- **External Database Links:** A link to other databased is provided for all the proteins individully. UniProtKB furnishes various features of the proteins with experimental and predicted information. PlasmoDB provides different knowledge at genomics and proteomics levels. Pfam provides the information about the protein family associated to the proteins. Links to external databases are provided wherever possible.

**
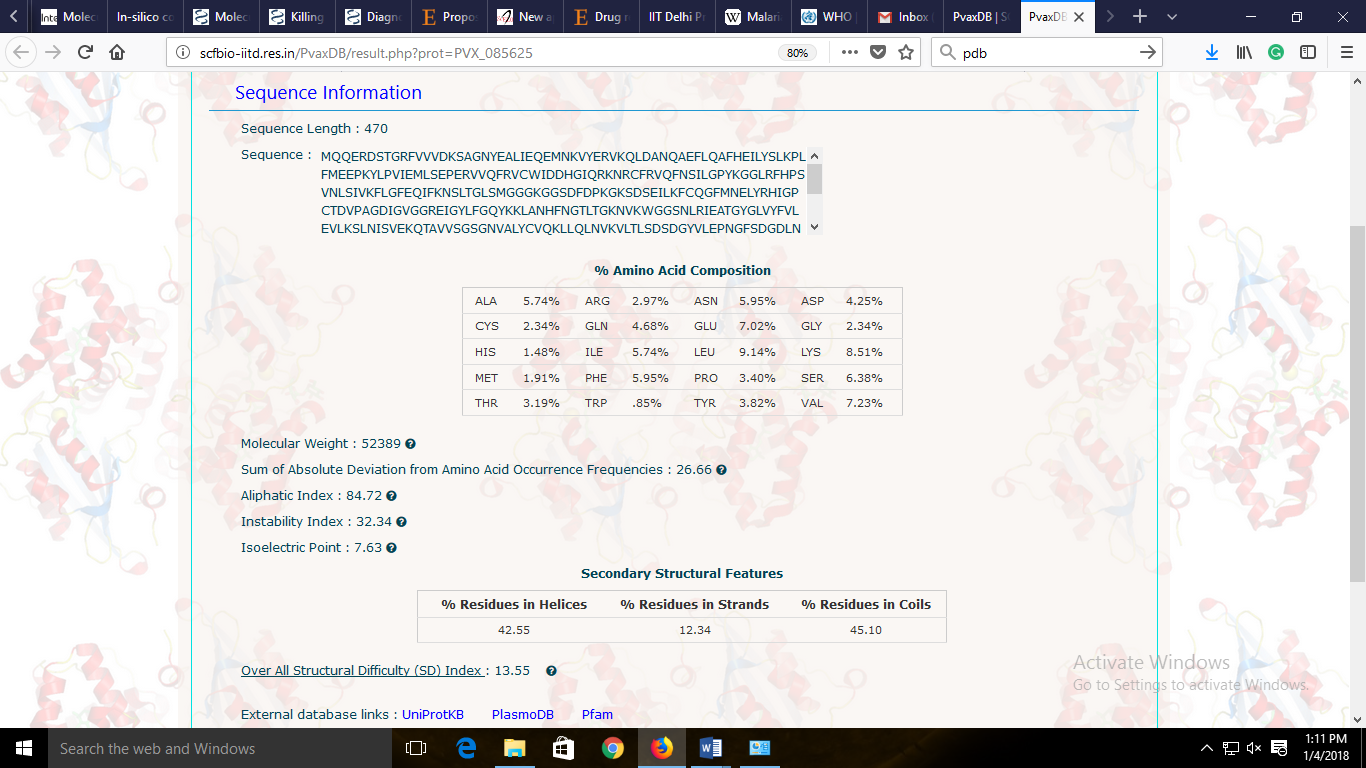
Figure S4**. A snapshot of individual sequence based information provided in PvaxDB for each protein.

**Step IV. Structural Information**

Further in PvaxDB, an extensive structural information for all the non-membrane proteins is provided. Protein structure prediction is performed via some of the state of the art methods viz. *BhageerathH^+^*, I-TASSER and RAPTORX. *BhageerathH^+^* and I-TASSER are *ab inito* / homology based hybrid methodology for structure prediction while RAPTORX performs homology based structure prediction.

A snapshot of predicted model structures corresponding to each methodology is provided on the result page along with the sequence coverage (start and end positions). Alternatively, user may visualize the model structures in three dimensional view by clicking on ‘View in Jmol’. The structures may be downloaded by clicking on download symbol next to method’s name. Also the images may be downloaded by simply clicking on them. Figure S4 shows a snapshot of PvaxDB webserver displaying structural information.

A comprehensive protein structure quality assessment is performed for all the model structures by using a metaserver approach, ProTSAV. The metaserver implements various structural features derived from individual tools to validate the accuracy of protein structures. The ProTSAV based quality assessment furnishes a depiction of normalized scores from individual tools (blue colored asterisk symbol) and an overall score (named as P-Score). The position of P-Score (blue dot on extreme right) in green, yellow, orange or red color indicates the overall quality of corresponding model structure. The structure with P-Score falling in green and yellow zone are considered as high and medium accuracy model structures respectively and can be used for further structure based studies. A detailed interpretation of quality assessment results is discussed using a case study (PVX_085625) for better clarity. Among multiple models, the model with lowest P-Score should be selected for further studies.

- **Case Study for PVX_085625**

A detailed explanation of quality assessment for PVX_085625 structure predicted by *BhageerathH^+^* is provided here. ProTSAV quality assessment, a collective result from different individual tools is provided. For any individual tools, the green region represent high accuracy model prediction (under 2Å predicted rmsd), yellow region represent medium accuracy model prediction (2-5Å predicted rmsd), orange region represents low accuracy model prediction (5-8Å rmsd) and red region represent bad structure prediction (beyond 8Å predicted rmsd). Additionally, a graphical representation for individual quality assessment viz. PROCHECK, ERRAT, Verify-3D and Naccess are provided. In case of PVX_085625, the predicted model structure showing the P-Score falling in green region validates high accuracy model structure. Likewise, user may interpret the structural information for predicted model structures via other prediction methods (I-TASSER and RAPTORX). Figure S5 depicts the result section for structural information of PVX_085625. Similar information is provided for all the non-membrane proteins addressed in PvaxDB. The individual quality assessment tools are discussed briefly to get an idea about the various parameters used for quality assessment. Also, Table S2 furnishes an overall view about quality assessment individual tools used as modules in ProTSAV metaserver along with their structural parameter used.

- **PROCHECK**: It evaluates the stereo-chemical quality of a protein structure by considering bond lengths, bond angles, main chain and side chain parameters, residue contacts, geometry, and distribution of backbone [torsion angles](http://www.sciencedirect.com/topics/biochemistry-genetics-and-molecular-biology/dihedral-angle) (Φ and Ψ) in [Ramachandran plot](http://www.sciencedirect.com/topics/biochemistry-genetics-and-molecular-biology/ramachandran-plot). An overall G-factor is calculated measures the extent of normal or unusual parameters. A higher value of G-factor is considered as indication of high accuracy model structure prediction. Also, higher percentage residues falling in allowed regions of Ramachandran Plot signifies better accuracy as shown in Figure S6(a).
- **ERRAT**: It distinguishes between correctly and incorrectly determined regions based on characteristic atomic interactions and provides an overall quality factor for the given structure. Based on the information derived from experimental structures, the threshold overall quality factor is considered to be 91% for medium resolution structures and 95% or above for high resolution (good) structures. A quality assessment of PVX_085625 by ERRAT for *BhageerathH^+^* is shown in Figure S6(b).
- **Verify-3D**: It performs the assessment of a protein tertiary structure by checking its compatibility with its amino acid sequence with a measure of 3D-1D Profile Score for each residue. Higher percentage of residues having average 3D-1D score more than 0.2 with a sliding window of 21 residues (falling in green region) indicates better protein structure prediction as shown in Figure S6(c)
- **
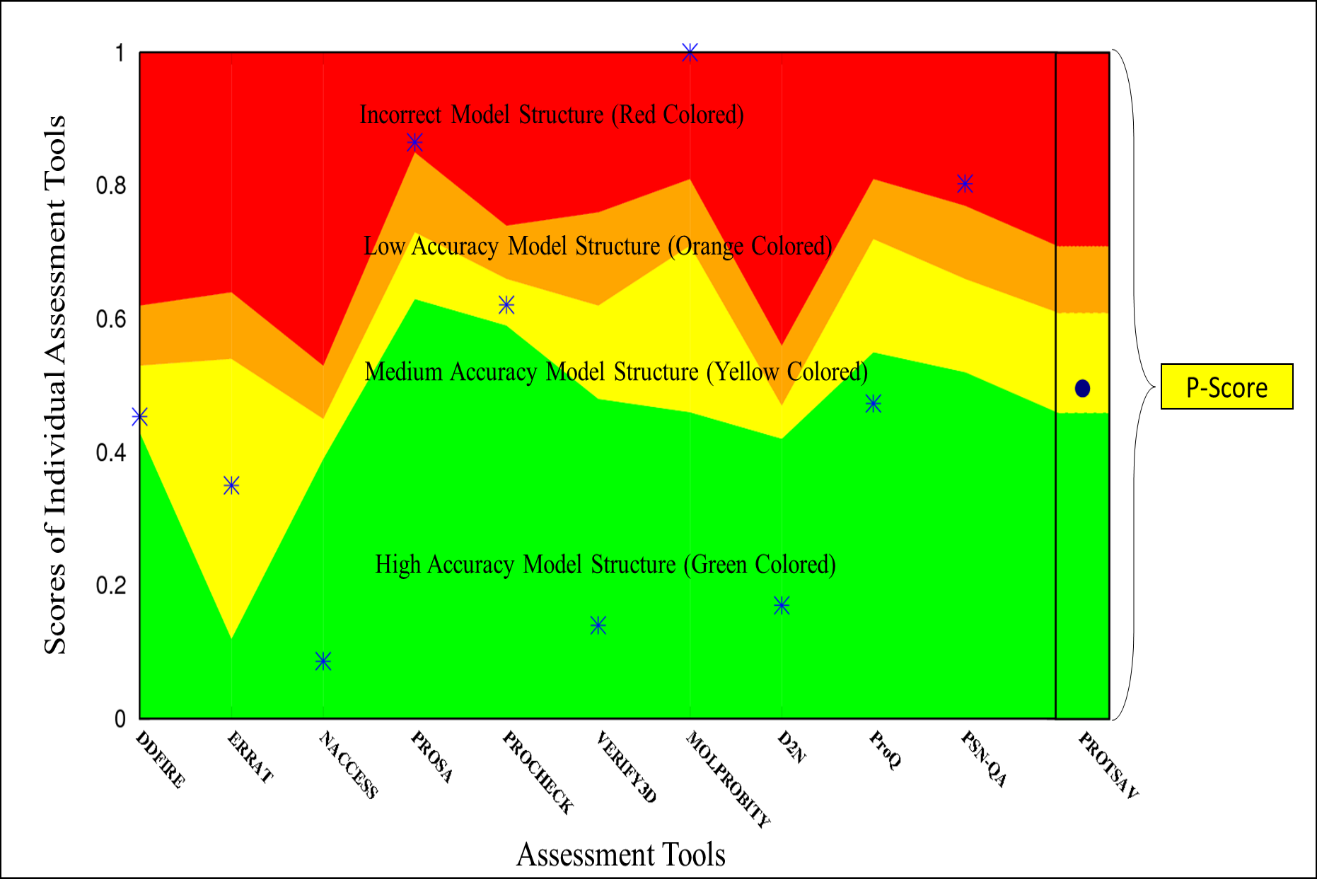
Naccess**: Naccess calculates the solvent accessibility of all atoms and residues with a defined probe size. High solvent accessible surface areas of a protein structure reflects bad quality of predicted model structure. A quality assessment of PVX_085625 by Naccess for *BhageerathH^+^* is shown in Figure S5(d) where green region represent correctly modeled residues.

**Figure S5.** An interpretation of ProTSAV metaserver based quality assessment.

**Table S2**. Different quality assessment tools/softwares and their quality assessment parameter where ‘Yes’ represents the implementation of the parameter in respective tool/software.

| Assessment Features | DFire | Errat | Naccess | ProSA | Procheck | Verify3D | D2N | ProQ |
| --- | --- | --- | --- | --- | --- | --- | --- | --- |
| Van der Waals Clashes |  |  |  | Yes |  |  | Yes |  |
| Contact Potential | Yes | Yes |  |  |  | Yes |  | Yes |
| Burial Preferences |  |  |  | Yes |  |  |  |  |
| Accessible Surface Area |  |  | Yes |  |  |  |  | Yes |
| Residue Packing |  | Yes |  |  |  |  |  |  |
| Globularity |  |  | Yes |  |  |  |  | Yes |
| Secondary Structure |  |  |  | Yes |  | Yes | Yes | Yes |
| Φ and ψ Distribution |  |  |  |  | Yes |  |  |  |
| Energy Based Scorings | Yes |  |  |  |  |  | Yes |  |
| Side Chain Packing |  |  |  |  | Yes |  |  |  |

**
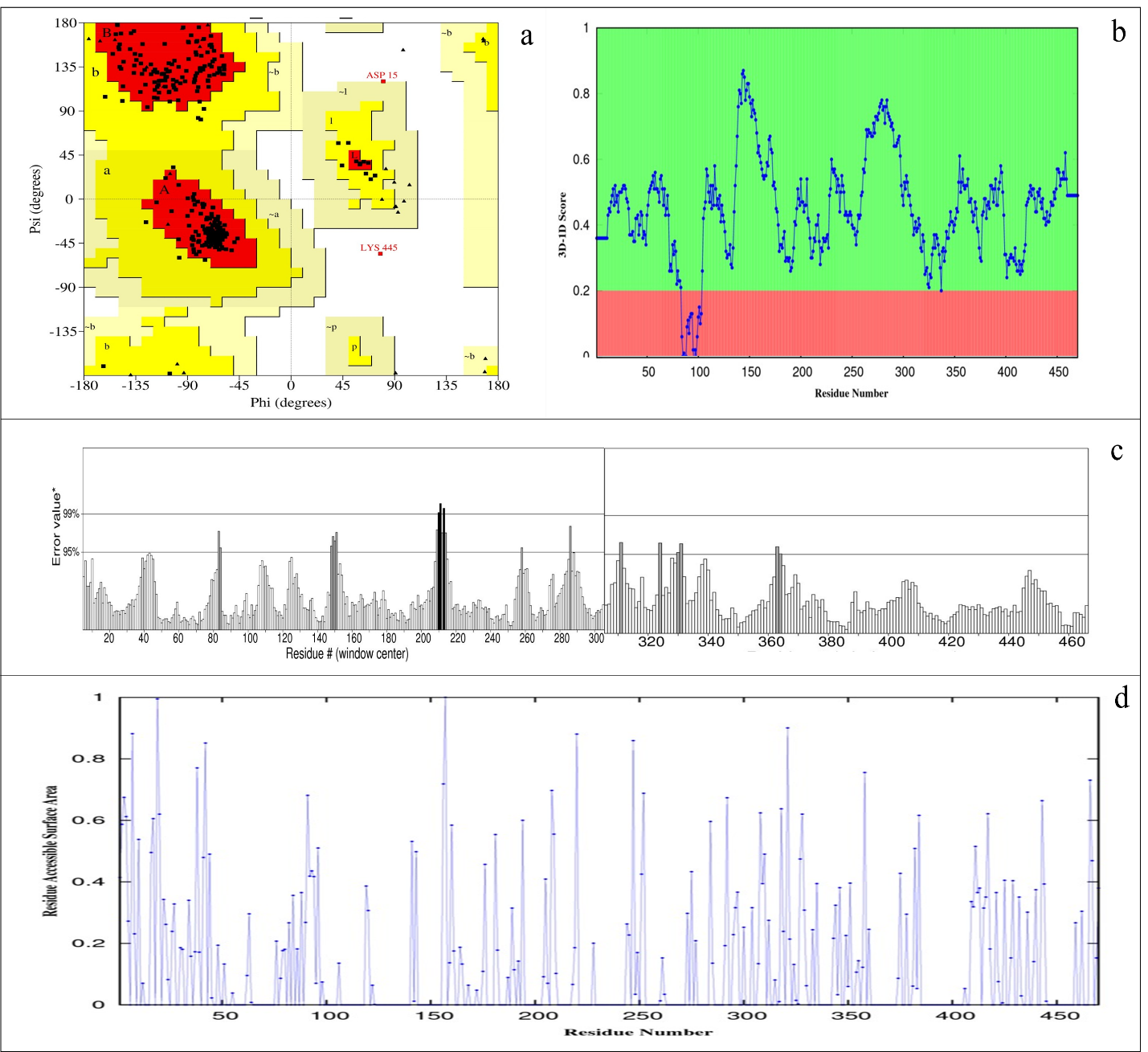
Figure S6.** Quality Assessment via different tools. (a) PROCHECK based backbone dihedral angles distribution in allowed (red and yellow) and disallowed regions of Ramachandran Plot, (b) Residue wise average 3D-1D scores of Verify3D, (c) ERRAT based quality assessment where black colored regions are erroneous, (d) Residue wise accessible surface area distribution calculated via Naccess and normalized to a 0-1 scale. Lower surface area is preferred.

**Step V. Ligand Binding Site**

The ligand binding site information is very useful in various structure based function annotations and drug discovery. For ligand binding site detection three different tools are used for a consensus view and more reliable predictions. These tools are discussed briefly in the following section.

- **LigSite:** It captures surface-solvent-surface events from an input protein structure via protein’s Connolly surfaces and identified pockets are ranked on the basis of the extent of conservation of surface residues involved.
- **F-pocket:** It is based on Voronoi partition and alpha sphere theory. The pocket ranking is performed via Partial Least Square fitting. It has been considered to track cavities on different, aligned protein structures. F-pocket accepts protein structure (pdb file format) and generates pocket information.
- **AADS:** It is an automated active site identification, docking and scoring protocol based on physico-chemical descriptors. It takes protein structure (in pdb file format) and predicts ten potential ligand binding sites.

In PvaxDB, the residues lining the predicted ligand binding site indicated in three dimensional view as well as in text as shown in Figure S7. For each method, at most four ligand binding sites are displayed on the ‘Ligand Binding Site’ section of result page. User may download the predicted model structures along with predicted ligand binding site information by clicking on the download symbol provided under the method’s name.

**
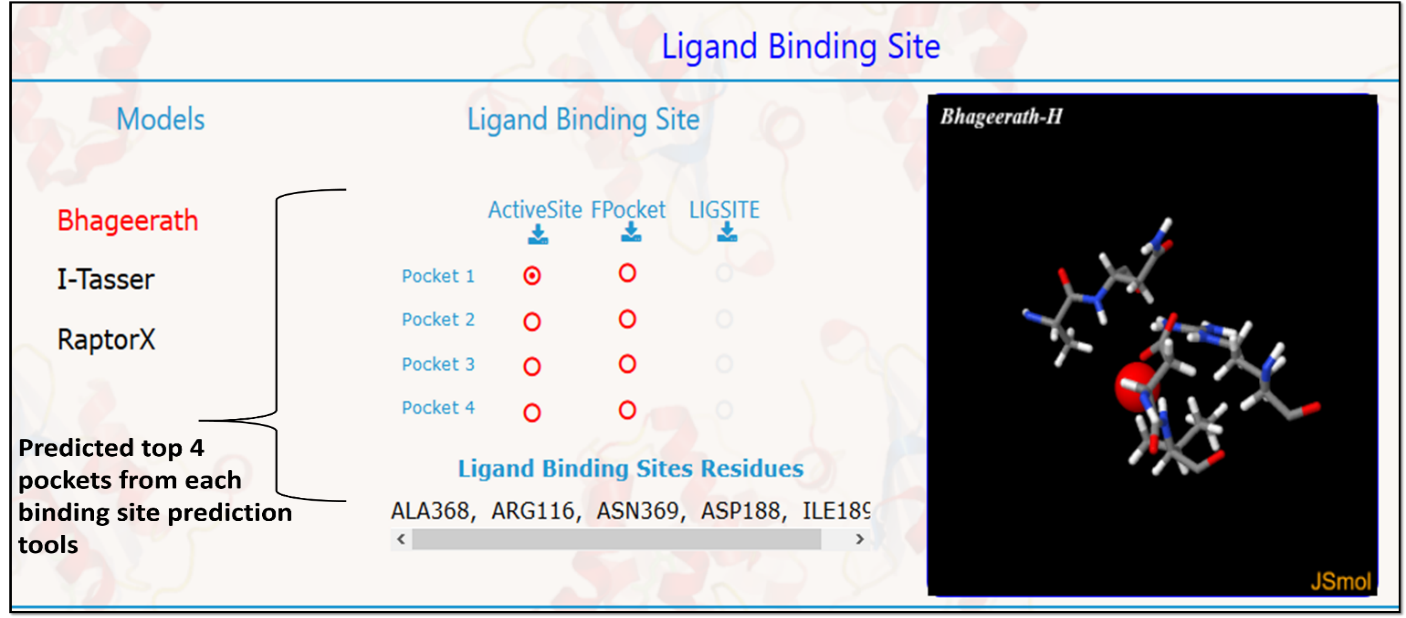
Figure S7.** A snapshot of ligand binding site information provided via PvaxDB. For each model structure at most 4 different binding sites are identified via each tool.

**Step VI. Functional Annotations**

In PvaxDB, function annotations of proteins is performed via InterPro, LocTree, SHIFTER and ProBiS. Additionally, the function information is also retrieved from UniProt (if available) and provided in PvaxDB. InterPro, LocTree and SIFTER perform function annotation from sequence information while ProBiS uses structural information. A brief summary of these methods is provided below.

- **InterPro:** It performs protein sequence analysis and classification by using prediction models or signatures compiled from different databases. It combines signatures from different databases into a single searchable resource, decreasing redundancy and helping users interpret their sequence analysis results. A standalone version of InterPro (InterProScan) is used to perform function annotations of protein sequences into corresponding gene ontology terms, protein families, domains, repeats, etc.
- **LocTree:** It is a support vector machine learning based hierarchical system for searching proteins of experimental localizations and function prediction. It accepts protein sequence as input and perform protein function prediction with a reliability score varying from 0 to 100 where 100 reflect most confident prediction.
- **SIFTER:** SIFTER (**S**tatistical **I**nference of **F**unction **T**hrough **E**volutionary **R**elationships) based function prediction implements a statistical approach using phylogenetic analysis for representing protein relationships. For function annotations in PvaxDB, protein sequences are used for performing SIFTER based function prediction. For an input protein sequence, SIFTER provides gene ontology terms and corresponding confidence scores.
-
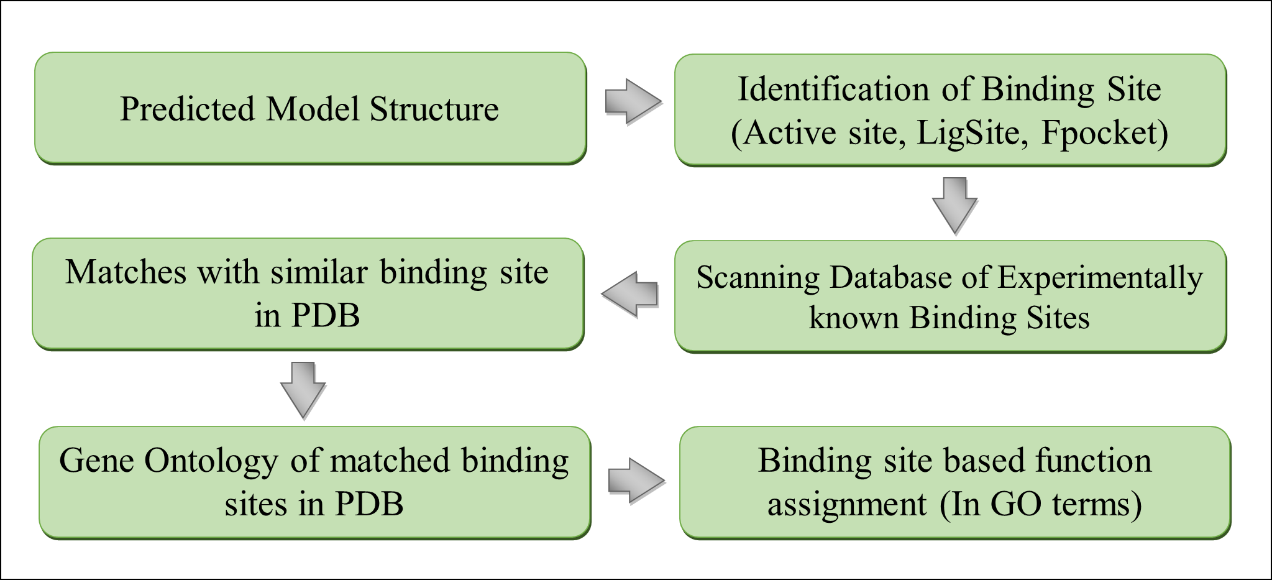
**ProBiS:** ProBiS is a protein structure surface conservation based similar protein binding sites detection tool. For an input protein structure or binding site, the method scans for similar binding site and provides pdb identifiers of matching binding sites. In PvaxDB, the gene ontology terms corresponding to these pdb file are used in assigning functions. A flowchart of ligand binding site based function assignment in PvaxDB is depicted in Figure S8.

**Figure S8.** A flowchart of structure based function assignment using ProBiS.

**
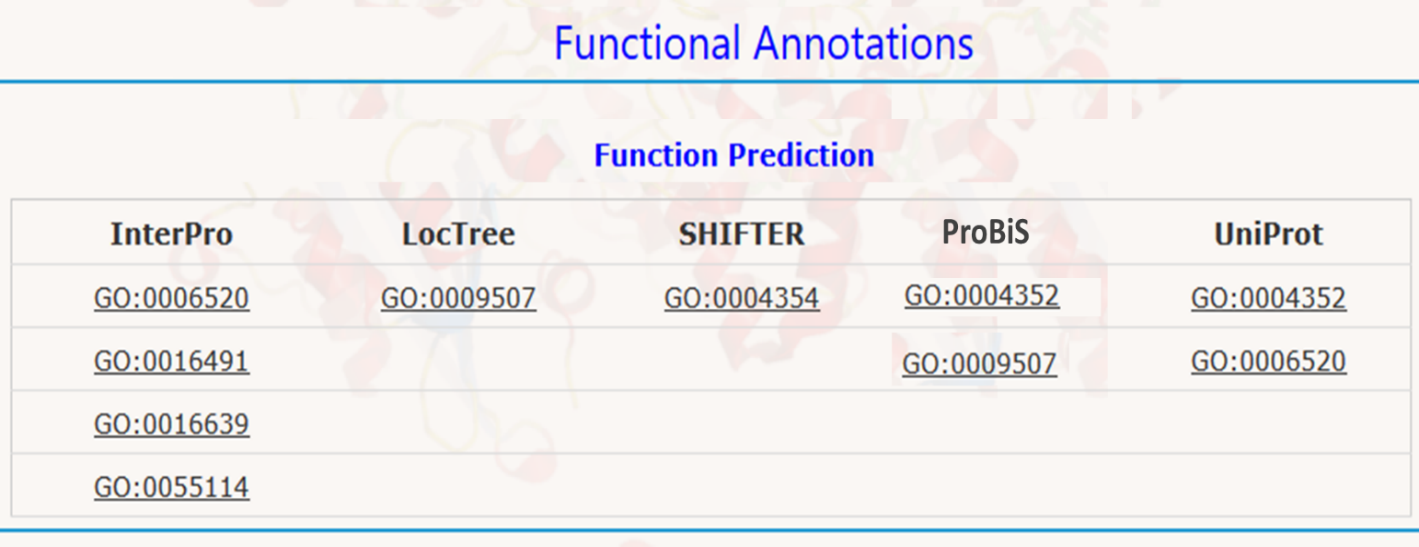
**A snapshot of PvaxDB for function annotations is shown in Figure S9.

**Figure S9.** A snapshot of function annotations performed in PvaxDB by different methods.
